# What could health technology assessment learn from living clinical practice guidelines?

**DOI:** 10.3389/fphar.2023.1234414

**Published:** 2023-08-24

**Authors:** Saskia Cheyne, Samantha Chakraborty, Samara Lewis, Sue Campbell, Tari Turner, Sarah Norris

**Affiliations:** ^1^ Faculty of Medicine and Health, University of Sydney, Sydney, NSW, Australia; ^2^ Australian Living Evidence Consortium, Cochrane Australia, Monash University, Melbourne, VIC, Australia; ^3^ Hereco, Sydney, NSW, Australia

**Keywords:** clinical practical guidelines, health technolgy assessment, living systematic review (LSR), regulatory policies and structures, reimbursement pathways, lifecycle HTA

## Abstract

A “living” approach to clinical practice guidelines is when the identification, appraisal and synthesis of evidence is maintained and repeated at an agreed frequency, with a clear process for when and how new evidence is to be incorporated. The value of a living approach to guidelines was emphasised during the COVID-19 pandemic when health professionals and policymakers needed to make decisions regarding patient care in the context of a nascent but rapidly evolving evidence base. In this perspective, we draw on our recent experience developing Australian and international living guidelines and reflect on the feasibility of applying living guideline methods and processes to a lifecycle approach to health technology assessment (HTA). We believe the opportunities and challenges of adopting a living approach in HTA fall into five key themes: identification, appraisal and synthesis of evidence; optimising the frequency of updates; embedding ongoing multi-stakeholder engagement; linking the emergence of new evidence to reimbursement; and system capacity to support a living approach. We acknowledge that the suitability of specific living approaches to HTA will be heavily influenced by the type of health technology, its intended use in the health system, local reimbursement pathways, and other policy settings. But we believe that the methods and processes applied successfully to guideline development to manage evidentiary uncertainty could be applied in the context of HTA and reimbursement decision-making to help manage similar sources of uncertainty.

## 1 Introduction

Health Technology Assessment (HTA) is a multidisciplinary process that uses explicit methods to determine the value of a health technology at different points in its lifecycle, for the purpose of informing decision-making that promotes an equitable, efficient, and high-quality health system (O’Rourke et al., 2020) It is a formal, systematic process for translating evidence into health policy. A full HTA typically includes the following domains: a description of the health problem and its current standard of care; a description of the proposed health technology or service; the comparative safety and effectiveness of the proposed health technology or service (with these elements typically framed using the PICO criteria—Population, Intervention, Comparator, Outcomes); an economic evaluation; a budget impact analysis; consideration of relevant organisational or implementation aspects; and consideration of relevant ethical, legal, and social aspects (EUnetHTA, 2016).

HTA is often reactive, occurring at a single point in time following initial regulatory approval or in response to regulatory changes (e.g., the expansion of approved indications) (CADTH, 2011; PBS Scheme, 2022). Full HTAs can take several months to years to complete. A lifecycle approach to HTA, whereby evidence is frequently incorporated and the HTA is dynamically updated, was first proposed in 2016 in order to more fully realise the benefits of innovations in healthcare (Husereau et al., 2016; Grammati et al., 2023). Since then a number of initiatives around the world have been exploring how a lifecycle approach to HTA can be implemented, for example, reassessments are performed by HAS and NICE, and conditional approvals exist in multiple countries such as the United Kingdom, the Netherlands and France (Ibargoyen-Roteta et al., 2022).

A lifecycle approach is even more relevant as agencies around the world are faced with assessing new, rapidl evolving classes of health technology, such as cell and gene therapies (Husereau et al., 2016). In this article, we share our recent experience developing and implementing methods and processes for Australian and international living guidelines and reflect on the opportunities and challenges of applying a living guideline approach to lifecycle HTA (Cheyne et al., 2023a).

## 2 Static *versus* living guidelines

The core methods for literature searching, evidence appraisal and synthesis are similar for living and partial updating of traditional (static) guidelines, but living guidelines involve a frequent and explicit approach to keeping the guidelines up-to-date. This approach includes frequent surveillance for newly published clinical studies, the prospective, ongoing incorporation of those studies into the evidence base, and the use of pre-agreed triggers for updating the corresponding evidence-based recommendations (Akl et al., 2017; Cheyne et al., 2023a; Cheyne et al., 2023b; Fraile Navarro et al., 2023; McDonald et al., 2023; Synnot et al., 2023) The criteria for selecting living topics are: clinical or policy priority of the question, important uncertainty in the existing evidence, and high likelihood of emergence of new evidence where the clinical/policy context is likely to change (Akl et al., 2017; Cheyne et al., 2023a). The frequency of updating a living topic is determined by the nature of the health problem, the flow of emerging evidence, the capacity of the evidence review team to search, screen and appraise new evidence, and the capacity of the Guideline Development Panel to meet and determine the implications of the new evidence (Cheyne et al., 2023b; McDonald et al., 2023) For example, searches for living COVID-19 guidelines were conducted on a daily basis during the height of the pandemic, whereas searches for living stroke guidelines are conducted every 3 months (Tendal et al., 2021; Hill et al., 2022).

The most tangible benefit of a living approach to guidelines is that evidence-based recommendations for clinical care retain their trustworthiness by remaining up-to-date. A less tangible (but no less important) benefit of a living approach is the way it changes the context for decision making: the knowledge that a decision can be revisited soon (typically in weeks or months) means that Guideline Development Panel members are more likely to make a decision on a recommendation in the face of uncertain evidence, rather than make no decision.

## 3 Similarities and differences between HTA and guidelines

Though intended for different purposes and audiences, HTA and clinical practice guidelines share core components, particularly those related to methods for the surveillance, appraisal, synthesis, and contextualisation of clinical and patient evidence (Guyatt et al., 2011; Higgins et al., 2022). Best practice in HTA and guideline development places an emphasis on early and ongoing multi-stakeholder involvement (Ibargoyen-Roteta et al., 2022). HTA and guideline development both rely on deliberative processes to translate evidence into recommendations for policy and practice.

However, there are important differences between HTA and guidelines. These differences arise from the fact that HTA has a broader scope than guidelines, is undertaken by industry as well as by government and non-profit organisations, is less transparent because of the inclusion of unpublished clinical data and commercially sensitive pricing information, and needs to comply with local regulatory and reimbursement pathways. This means that it is more straight-forward to change a guideline recommendation than it is to change an HTA decision. It also means that it cannot be assumed that the methods and processes applied in living guidelines are directly transferable to all HTA in all settings.

Despite the differences, HTA and guideline development are interdependent activities that draw from the same knowledge base: HTA often relies on guidelines to define current treatment pathways and comparators; and guidelines need to be cognisant of the regulatory and reimbursement status of treatments they recommend. The need for harmonisation of HTA and guidelines (e.g., as undertaken by NICE in the United Kingdom) is an important area of health services research and has been described by others, but is not the focus of the current article (Schünemann et al., 2022). Early multi-stakeholder dialogue frameworks allow for health technology developers to incorporate advice from HTA agencies in their health technology planning and to directly address uncertainty during technology development (Ibargoyen-Roteta et al., 2022; Hogervorst et al., 2023).

## 4 Opportunities and challenges in adopting a living guideline approach for HTA

We see a number of opportunities and challenges for adopting a living guideline approach in HTA (Table 1). The living guideline approaches most obviously suited to HTA relate to the methods of evidence assessment. The tools to support standard and living systematic reviews are advancing rapidly, and the potential for these to be incorporated within HTA methods have been described by others (Grammati et al., 2023; Thokala et al., 2023). To date, most evidence review within living guidelines has been limited to randomised controlled trials (RCTs) of interventions. By contrast, HTAs often include diagnostic, prognostic, economic and epidemiological questions, in addition to intervention questions, and the inclusion of non-randomised controlled data such as longer term safety evidence from observational studies or registry data. HTA is now often reliant on single-arm trials and “Real World Evidence” and a number of organisations are exploring the use of such data in HTA (HAS Sante, 2021; NICE, 2022; Bakker et al., 2023). It should be feasible, though, for a living approach to be adopted across all types of evidence searching that occur within an HTA. For example, living guidelines for COVID-19 diagnostics for antigen, serology and molecular testing (Hanson et al., 2021) and living systematic reviews are frequently conducted on these types of questions (Wynants et al., 2020).

**TABLE 1:** Opportunities and challenges for adopting a living guideline approach for HTA.

Opportunities	Challenges
1. Evidence identification, appraisal and synthesis	
• Preparing clinical evidence syntheses in standardised and shareable formats to minimise duplication of effort across agencies. (e.g., the use of GRADE and MAGIC for living guidelines has enabled the sharing of Evidence Profile tables between countries)	• How and when to include unpublished clinical evidence
	• How to include evidence for diagnostic, prognostic, economic, and epidemiological questions
	• How to store data securely whilst enabling sharing
	• Copyright restrictions around data extracted from published evidence
2. Optimising the frequency of updates	
• More frequent updates of the evidence could resolve uncertainty regarding the technology, care pathways, patient group, uptake, market share, or economic modelling, especially where conventional evidentiary standards have not been met.	• Reimbursement and procurement systems may not be designed for frequent changes in pricing for a health technology
3. Embedding multi-stakeholder engagement	
• Early identification and ongoing dialogue with all relevant stakeholders (as occurs with a living Guideline Development Panel) would support planning and scoping for HTA	• How to facilitate effective engagement and communication between stakeholders with different perspectives or priorities (payers/government, industry, regulatory bodies, healthcare providers, healthcare professionals, patients)• How to share commercially sensitive information amongst this wider group of stakeholders
4. Linking the emergence of new evidence to reimbursement	
• Re-evaluation and value-based renegotiation in response to new evidence (especially where conditional funding decisions have been made) • Decision-makers may be more inclined to provide conditional reimbursement for technologies if they are confident that decisions can be reversed if no definitive evidence of effectiveness emerges	• Pricing negotiation and/or the implementation of new pricing agreements can be protracted and may negate any reductions in time to market access • The framework for renegotiation of pricing needs to allow for price increases as well as price decreases or disinvestment (either complete de-adoption of technologies that are not clinically effective or restrictions to ensure cost-effective use)
5. System capacity to support a living approach	
• More certainty in the timing and scope of HTA which enables better workforce planning for those undertaking the HTA.	• Fixed schedules for reimbursement decision-making • Regulatory or legislative changes may be required to compel technology developers to provide the required data • Having sufficient methodological capacity on hand to ensure the timely inclusion of new evidence as it emerges

Similar and additional factors are likely to determine the frequency with which HTA literature searches can be updated, including a combination of the capacity of HTA teams to undertake more frequent searching, and the frequency with which the respective decision-making entities can meet to adjudicate on the new evidence. One issue to be mindful of is that the frequency of updating decisions does not outpace the ability of the health system to respond. The frequency of guideline recommendation revisions is effectively limited by the ability of healthcare providers to modify local protocols and standards for care. However, the frequency of reimbursement revisions will be limited by the frequency at which decision-makers can consider updates, and the frequency with which pricing and supply contracts between industry and payers can be varied.

**FIGURE 1:**
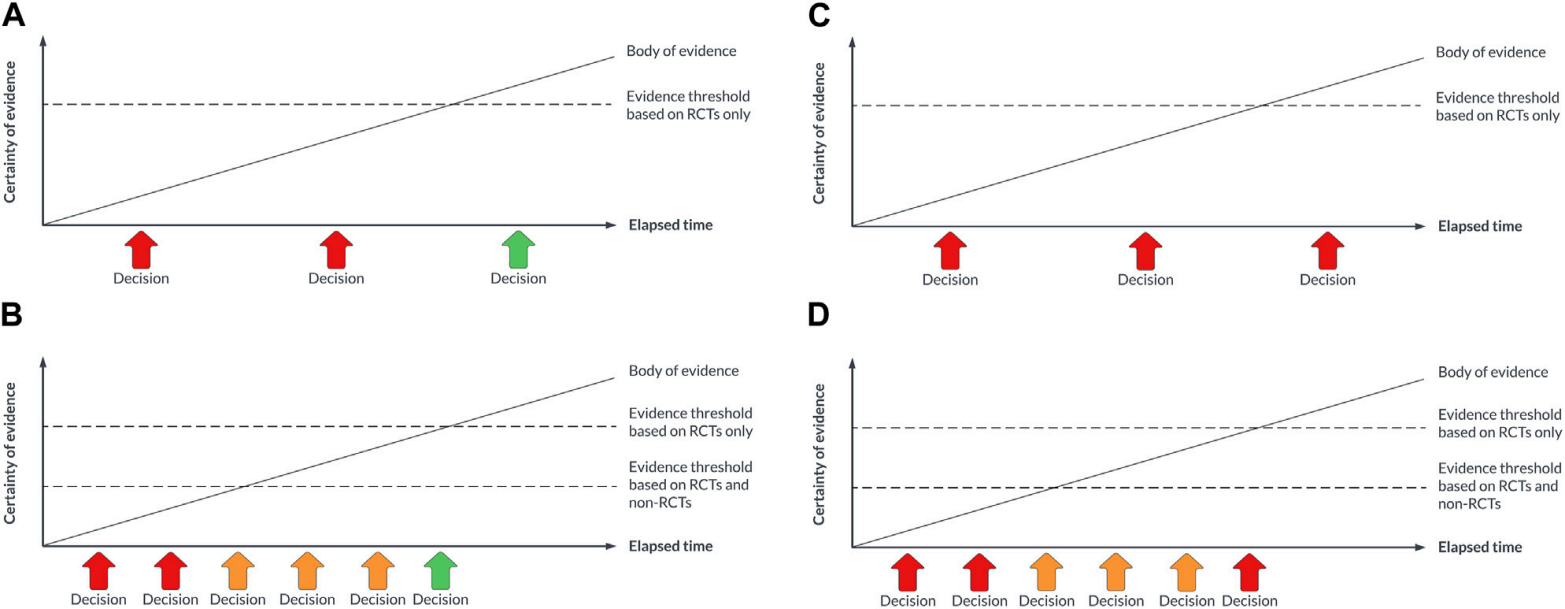
**(A)** Decision-making with a traditional approach to HTA for a technology that is eventually demonstrated to be safe, effective, and cost-effective. **(B)** Decision-making with a living approach to HTA for a technology that is eventually demonstrated to be safe, effective, and cost-effective. **(C)** Decision-making with a traditional approach to HTA for a technology that is eventually not demonstrated to be safe, effective, and cost-effective. **(D)** Decision-making with a living approach to HTA for a technology that is eventually demonstrated to not be safe, effective, and cost-effective.

As is for guidelines, it is unlikely that all HTA questions will be suitable for a living approach. Given the organisational changes that would be required to support a living approach to HTA, agencies may wish to focus on technologies that promise a high benefit-to-risk ratio, where the usual levels of RCT evidence are not available and where the cost implications are significant (e.g., cell and gene therapies), or where the pace of technological innovation is very high (e.g., digital health technologies) or the policy context is changing rapidly (e.g., the use of AI in diagnostics). In these situations, it should be possible to adopt a concept known as early multi-stakeholder dialogue, which is a prospective or intentional approach to HTA where manufacturers, healthcare providers, clinicians and payers pre-agree i) the measures of most relevance for the technology and the population(s) of interest, and ii) how the pricing of the technology will vary based on those measures (Schünemann et al., 2022). Any non-RCT data informing the decision-making will need to be considered trustworthy by HTA agencies and the payer (NICE, 2022). An illustration of this approach is shown in Figure 1.

Conditional marketing authorization pathways or lifecycle approaches to HTA have been introduced for cancer drugs and for digital health technologies (Sabry-Grant et al., 2019). These pathways incorporate some elements of a living approach by allowing the flexibility to provide temporary access to treatments in limited circumstances as more evidence accumulates (Hoekman et al., 2015; Regier et al., 2022). The use of a living approach here may provide the necessary flexibility in a more robust way, with an intention from the outset to continue updating the HTA with new evidence until a higher degree of certainty is reached, or to revise or rescind an access decision if reliable evidence of a net positive effect is not eventually obtained.

A living approach to HTA could decrease research waste and duplication of effort. The sharing of evidence summaries already happens in clinical practice guidelines (NICE, 2021), and there are steps towards this happening between HTA agencies in Canada, Europe, and Australia, (PBS, 2022; Hogervorst et al., 2023), however in reality the confidentiality of pricing arrangements, and the potential for price lowering or disinvestment at future reassessments, will limit the extent of such sharing (Thokala et al., 2023).

## 5 Discussion

In this perspective we have discussed the aspects of HTA that are most amenable to a living approach and where living guideline evidence translation methods or processes can be transferred to HTA. We also highlight what, in our view, is needed to support a transition to living HTA (Box 1).

Box 1 What is needed to optimise the impact and reliability of a living approach to HTA.

1. Development and/or testing of methods for the continuous updating of non-RCT evidence.
2. Pilot studies for different technologies for different clinical purposes, to understand what works, what does not work, and why, and the importance of context (i.e., the local health system, approaches to HTA, and health system financing).
3. Agreement on the HTA scenarios where a living approach is likely to optimise market access, defined as a combination of shorter time to market, with acceptable mitigation of safety risk to patients, and acceptable cost and cost-effectiveness.
4. Agreement on the policy levers that will be required to support partial or full disinvestment if technologies do not live up to their promise.
5. Practical guidance on the organisational and resourcing requirements for living HTA, and how to transition from reactive HTA at a single point in time to responsive HTA throughout the life-cycle of a technology.

The iterative nature of a living process allows for more nuance in the face of uncertainty, and a willingness to support innovation at early stages, knowing that decisions will be revisited and revised as new evidence emerges. It could give decision-makers comfort in making early conditional decisions for a technology/service, instead of what might otherwise be a “no” decision in the face of uncertainty. The “secret sauce” of a living guideline approach is the organisational infrastructure and collaborative culture that needs to be put in place to support it. It requires a commitment on the part of the guideline developer to provide ongoing funding to resource continuous evidence review activities, and a standing Guideline Development Panel to deliberate on new evidence as and when it emerges. Although a lot of HTA activity is undertaken as ‘one off’ evidence reviews, it should be possible for industry and HTA agencies to re-orient some (if not all) of their resources to a framework that supports the ongoing incorporation of new data (e.g., from health administrative systems or clinical quality registries). There is also additional efficiency to be gained by aligning the methods and timing for living guidelines and lifecycle HTA.

HTA agencies are under increased pressure to provide patients with early access to promising health technologies, while accounting for the often-incomplete picture of clinical and economic impact of a new treatment during its initial technology assessment. Often, the evidence available at the time of the first HTA is limited, and decision uncertainty may be reduced with longer term data from trials, observational and registry data. At the level of evidence review methods, further innovation and testing of living methods is required for study designs other than RCTs and for non-intervention questions, particularly given the drive for HTA to rely more on innovative clinical trial designs (e.g., platform and adaptive trials). Living HTA could expand the approaches employed by living guidelines in two key ways: By 1) including pricing/cost considerations in the prioritisation criteria for living topics, and 2) exploring how living searches for economic and epidemiological data could feed in to economic evaluations and budget impact analyses. The policy challenges of adopting a living approach in HTA are more significant than for a living approach to guidelines: the benefits of earlier patient access to treatments need to be balanced against the potential for making “wrong” decisions—reimbursing technologies that do not end up being as safe, effective and/or cost-effective as anticipated. This highlights the importance of developing trust between stakeholders *before* living approaches are implemented, and finding a balance between policy levers that “push” (e.g., requiring developers to provide data on their technology) and “pull” (e.g., earlier market access) towards a living approach.

The introduction of the living approach may result in the ability to create a more harmonious and streamlined process between both HTA and guidelines. In this perspective we have illustrated the HTA domains where living guideline evidence translation methods or processes are directly transferable to HTA, additional aspects of HTA where a living approach is likely to be suitable (but where methods and processes still need to be developed); and aspects of HTA that are unlikely to be suitable for a living approach. However, our experience is limited by primarily conducting living guidelines and HTAs in an Australian context. Pilot case studies are needed that 1) describe the experience of introducing different living methods or processes within different HTA scenarios, 2) determine benefits and challenges of these approaches, 3) further develop methods for those areas of living methods that are specific to HTA, such as economic analysis, and 4) place these experiences within the local policy context so that broader themes can be identified regarding the suitability of living methods and processes for HTA in different countries.

## Data Availability

The original contributions presented in the study are included in the article, further inquiries can be directed to the corresponding author.
